# The Effect of Laser Trabeculoplasty on Posture-Induced Intraocular Pressure Changes in Patients with Open Angle Glaucoma

**DOI:** 10.1371/journal.pone.0147963

**Published:** 2016-01-25

**Authors:** Jee Myung Yang, Mi Sun Sung, Hwan Heo, Sang Woo Park

**Affiliations:** 1 Department of Ophthalmology, Chonnam National University Medical School and Hospital, Gwang-ju, Republic of Korea; 2 Center for Creative Biomedical Scientists, Chonnam National University, Gwang-ju, Republic of Korea; University of California San Diego, UNITED STATES

## Abstract

**Purpose:**

To investigate the effect of argon laser trabeculoplasty (ALT) on posture-induced intraocular pressure (IOP) changes in patients with open angle glaucoma (OAG).

**Methods:**

Thirty eyes of 30 consecutive patients with OAG who underwent ALT were prospectively analyzed. The IOP was measured using Icare PRO in the sitting position, supine position, and dependent lateral decubitus position (DLDP) before ALT and at 1 week, 1 month, 2 months, and 3 months after ALT.

**Results:**

Compared to the baseline values, the IOP in each position was significantly decreased after ALT (all *P* < 0.001). During follow-up, the mean percentage of IOP reduction was similar in the sitting and supine positions, but was significantly lower in DLDP than in the sitting or supine positions (all *P* < 0.05). In terms of postural IOP changes, the IOP in the supine position and DLDP was significantly higher than that in the sitting position at the same time points during the follow-up period (all *P* < 0.001). The difference between the IOP in the supine position and DLDP during follow-up was significant (all *P* < 0.001). The extent of IOP differences between any positions did not show significant changes during the follow-up period (all *P* > 0.05).

**Conclusions:**

ALT appears to be effective in lowering the IOP in various body positions, but the degree of this effect was significantly lower in DLDP. In addition, ALT seemed to have limited effects on posture-induced IOP changes.

## Introduction

Elevated intraocular pressure (IOP) has long been considered an important risk factor for the onset and progression of glaucoma [1–3]. Not only the elevation of IOP, but also the IOP fluctuation is considered one of the important factor for the progression of glaucoma [4–8].

The postural change from the sitting to supine or lateral decubitus position (LDP) can increase IOP significantly [9–11]. IOP fluctuations due to postural changes have been suggested to be closely related to structural and functional deterioration in glaucomatous eyes [10,12–14]. Kim et al.[15] reported that the preference of the open angle glaucoma (OAG) patients for LDP during sleep is associated with greater functional deterioration and visual field loss, which are more pronounced in the dependent eye than in the nondependent eye. Therefore, when it comes to glaucoma management, IOP fluctuation due to postural changes should be taken into account and the ideal strategy would be to achieve target pressure with minimal IOP fluctuation.

Argon laser trabeculoplasty (ALT) popularized by Wise and Witter [16] is an effective therapeutic option to lower IOP in OAG patients. It is as effective as hypotensive medical treatment, and has become the treatment of choice in patients refractory to maximal tolerated medical treatment [17,18]. It may be an appropriate alternative to glaucoma filtering surgery and is hoped to postpone or even eliminate the need for invasive surgical intervention.

In terms of IOP fluctuation, there have been several studies on posture-induced IOP changes in glaucoma patients after filtering surgery [19–21]. However, little attention has been given to posture-induced IOP changes in glaucoma patients after laser trabeculoplasty and most studies mainly focused on the 24-h IOP-lowering effect of laser trabeculoplasty [22–25].

Singh et al.[26] have studied the posture-induced IOP changes following ALT in 29 glaucomatous eyes. They concluded that ALT produced little effect on the posture-induced IOP changes. However, their study did not address statistical analysis of the data and IOP changes in dependent LDP (DLDP), which might play a significant role in the progression of glaucoma. The aim of the present study was to investigate the effect of ALT on the posture-induced IOP changes over time in OAG patients.

## Methods

### Subjects

A prospective interventional study was conducted in accordance with the Declaration of Helsinki. Written informed consent was obtained from all subjects, and the protocol was approved by the Institutional Review Board of Chonnam National University Hospital. Subjects who underwent ALT between July 2013 and February 2014 at the Department of Ophthalmology, Chonnam National University Medical School and Hospital were enrolled.

Baseline examinations included variables such as age, sex, best-corrected visual acuity, manifest refraction measured using an automated refractometer (KR8900; Topcon Corporation, Tokyo, Japan), IOP measurement with Goldmann applanation tonometry (GAT), slit-lamp biomicroscopy, anterior chamber angle examination by gonioscopy, central corneal thickness measurement by ultrasonic pachymetry (UP-1000; Nidek, Gamagori, Japan), axial length measurement by an A-scan biometer (Teknar Ophthasonic A-scan III; Teknar Inc., St Louis, MO, USA), optic nerve head and retinal nerve fiber layer (RNFL) examination with disc photography and red-free RNFL photography, Swedish interactive threshold algorithm standard 30–2 perimetry with a Humphrey field analyzer (Carl Zeiss Meditec, Inc., Dublin, CA, USA). The computed refractive error was converted into the spherical equivalent refractive error for statistical comparison. Inclusion criteria were as follows: (1) age ≥ 18 years; (2) open angle confirmed by gonioscopy; (3) typical glaucomatous optic disc damage with glaucomatous cupping and loss of the neuroretinal rim confirmed by fundus examination; (4) glaucomatous visual field (VF) defect corresponding to optic disc damage; (5) progressive or anticipated optic nerve damage or visual field loss despite maximal tolerated medical treatment (≥ three different topical hypotensive medications or insufficient IOP control with maximal tolerated hypotensive medications); (6) best-corrected visual acuity ≥ 20/40; and (7) spherical equivalent refractive error between −6.0 and +3.0 diopters and cylinder correction within 3.0 diopters, excluding patients with high myopia who could show greater IOP fluctuations [10,27,28]. Typical glaucomatous optic disc damage was defined as the vertical cup-to-disc ratio ≥ 0.7 or ≥ 0.2 asymmetry between eyes, or the presence of localized neural rim notching, excavation, or generalized loss of the neural rim [29]. Criteria for glaucomatous VF defects were as follows: ≥ 3 significant (*P* < 5%) non-edge contiguous points (including ≥ 1 at the *P* < 1% level) on the same side of the horizontal meridian in the pattern standard deviation (PSD) plot, confirmed by ≥ 2 consecutive examinations [29]. Patients were excluded if they had a history of glaucoma other than OAG, secondary glaucoma (including exfoliation glaucoma, pigmentary glaucoma, and uveitis glaucoma), ocular trauma, ocular surgeries including prior ALT, ocular diseases, or general medical conditions affecting the optic nerve or retina. The treatment eyes were selected according to inclusion and exclusion criteria, and if both eyes were treated, only the right eye was chosen for data analysis.

### Measurement of IOP

Our protocol included IOP measurement before treatment and at 1 week, 1 month, 2 months, and 3 months after ALT. All the IOP measurements were obtained between 4 PM and 6PM. The IOP in the sitting position, supine position, and DLDP was measured with the Icare rebound tonometry (Icare PRO; Icare Finland Oy, Helsinki, Finland) by a single examiner (J.M.Y) who was blinded to the patients’ characteristics. In the DLDP, the eye scheduled for ALT was located in the dependent position. When measuring supine position, the probe was place in a vertical position; the Icare PRO has been designed to hold the probe in place which prevents dropping. The tonometer has an intrinsic sensor that detects vertical inclination; an arrow appears in the display indicating the right vertical position to measure supine position [30]. The IOP was measured after maintaining each position for 10 min in the following sequence: sitting position, supine position, and DLDP. To measure the IOP in the supine position and DLDP, a soft pillow was placed under the head so that the head was maintained parallel to the bed. For each position, 3 consecutive sets of measurements were made (6 measurements for each set). The average values of each set were calculated automatically, and the mean values obtained from 3 consecutive set of measurements in each position were used for statistical analyses.

### ALT procedure

ALT was performed under topical anesthesia (proparacaine hydrochloride 0.5%) with the patient sitting in front of the slit lamp. All procedures were performed by a single surgeon (S.W.P). A Goldmann 3-mirror lens was placed on the eye with 1% methylcellulose to visualize the angle structures and the aiming beam was focused onto the junction of the pigmented and non-pigmented trabecular meshwork. On average, 50 applications of adjacent non-overlapping 50-μm spots with a 0.1-s pulse duration were performed to the inferior 180° of the trabecular meshwork. From 700 to 1200 mW of energy was delivered depending on the level of trabecular meshwork pigmentation [31–33]. Apraclonidine 0.5% (Iopidine; Alcon Laboratories, Fort Worth, TX, USA) was applied before and after the procedure. Patients were instructed to instill Rimexolone 1% (Vexol; Alcon Laboratories) in the treated eye 4 times a day for 1 week. All participants were maintained on their individual IOP-lowering medication regimen, which they had been used before ALT, throughout the study period.

### Statistical Analysis

Statistical analyses were performed using SPSS software version 18.0 (SPSS, Chicago, IL, USA). Data are presented as mean ± standard deviation (SD). The normality of variable distribution was verified by the Kolmogorov-Smirnov test. Correlation between the IOP measurements obtained by GAT and Icare PRO at sitting position was assessed using Pearson’s correlation test. To assess the IOP changes during the follow-up period, paired *t*-test and repeated measure analysis of variance (ANOVA) with post hoc Bonferroni correction for multiple comparisons were used [21]. The assumption of sphericity was tested with the Mauchly’s test and if the data violated the sphericity assumption, Greenhouse—Geisser correction was applied. Based on the prior pilot study, a sample size calculation determined that 30 patients would be required to detect an anticipated IOP difference between positions of 2.0 mmHg at a standard deviation of 3.8 mmHg with a power of 80%. *P* < 0.05 was considered statistically significant.

## Results

A total of 30 eyes of 30 patients with OAG met the criteria for inclusion in this study. The baseline characteristics of the study patients are listed in Table 1. The mean age was 52.0 ± 12.4 years (range, 27–73). There were 23 (76.7%) men and 7 (23.3%) women. The baseline IOP measured by GAT at sitting position was 18.9 ± 2.8 mmHg, which showed strong correlation with the Icare PRO (r = 0.885, *P* < 0.001). All patients were on maximal tolerated medical treatment before ALT.

**Table 1:** Baseline Characteristics of Participants.

Number of eyes (patients)	30 (30)
Laterality (right:left)	15:15
Age, years	52.0 ±12.4 (27–73)
Gender, n (%)	
Male	23 (76.7)
Female	7 (23.3)
IOP, mmHg*	18.9 ± 2.8 (14.8–25.0)
SE, D	-0.7 ± 1.4 (-4.0 to +2)
BCVA, LogMAR	0.1 ± 0.1 (0.0–0.3)
Central corneal thickness, μm	544.3 ± 25.6 (483–588)
Axial length, mm	23.5 ± 1.2 (21.6–25.7)
HVF	
MD, dB	-10.0 ± 8.1 (-3.0 to -26.9)
PSD, dB	7.3 ± 4.6 (2.5–16.6)
VFI	76.0 ± 25.2 (35–98)
Number of ocular hypotensive medications	2.1 ± 0.8 (1–3)

BCVA, best-corrected visual acuity; D, diopters; HVF, Humphrey visual field; IOP, intraocular pressure; LogMAR, logarithm of the minimal angle of resolution; MD, mean deviation; PSD, pattern standard deviation; SE, spherical equivalent; VFI, visual field index. Data were expressed as mean ± standard deviation (range)

* Measured by Goldmann applanation tonometer

**Fig 1:**
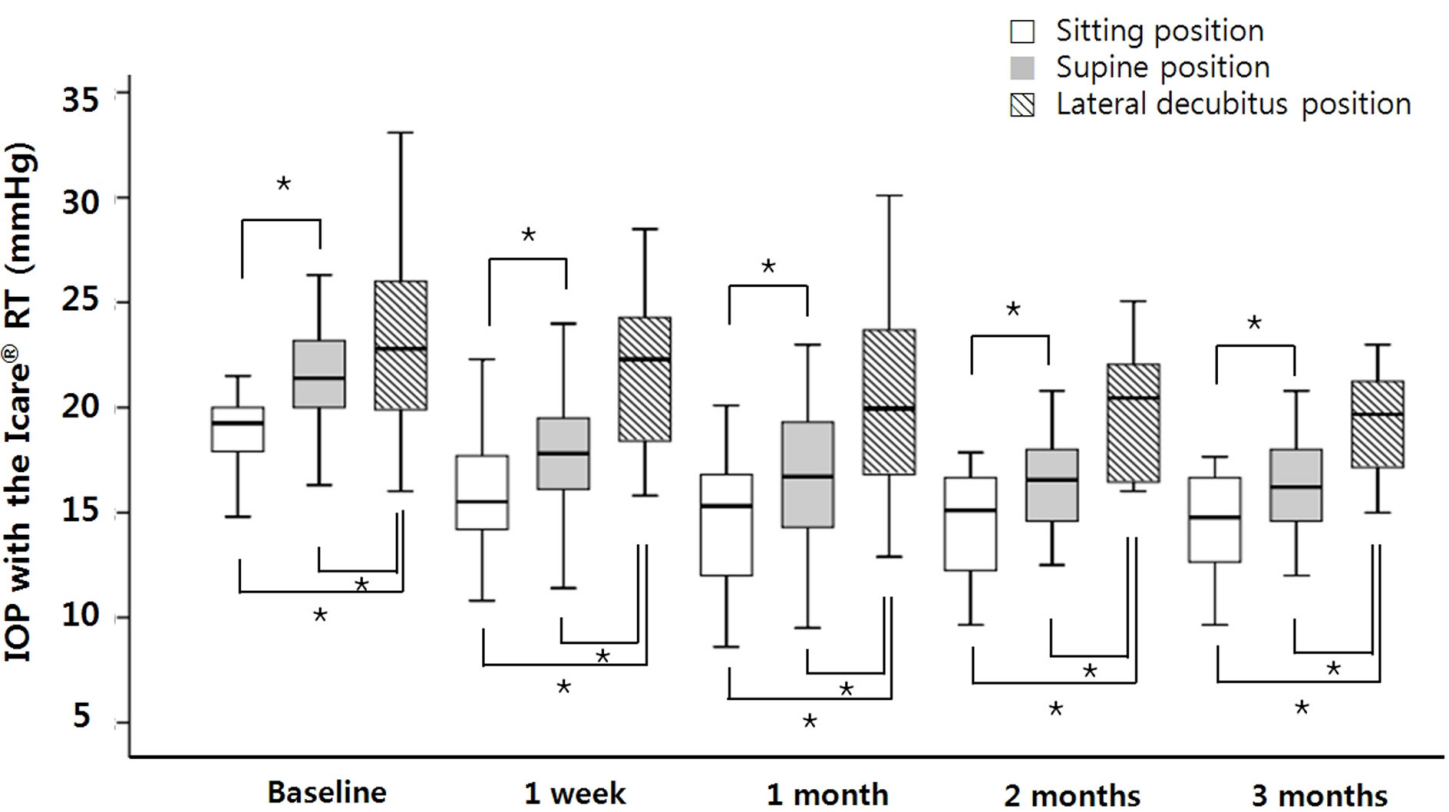
Posture-induced intraocular pressure (IOP) changes before and after ALT over a 3-month follow-up period. Compare to baseline value, IOPs in each position were significantly decreased at every time point of the follow-up period (*P* < 0.001; repeated measures analysis of variance followed by Bonferroni post-hoc test). Compare to sitting position, the IOP in the supine position and dependent lateral decubitus position (DLDP) were significantly higher at every time point of the follow-up period (*P* < 0.001; paired *t*-test). Also, the IOP between supine position and DLDP during follow-up were significantly different (*P* < 0.001; paired *t*-test). Box plots illustrate the median (50th percentile) as a black center line and the edges of the box are the 25th and 75th percentiles. The whiskers extend to largest and smallest values within 1.5 interquartile range. *P < 0.05

Before ALT, the mean baseline IOP measured by Icare PRO was 19.0 ± 2.4 mmHg in the sitting position, 21.2 ± 2.9 mmHg in the supine position, and 23.8 ± 4.6 mmHg in DLDP. After ALT, the mean IOP values decreased to 15.6 ± 2.7 mmHg (1 week), 14.8 ± 3.4 mmHg (1 month), 14.3 ± 2.7 mmHg (2 months), and 14.1 ± 2.6 mmHg (3 months) in the sitting position, 17.8 ± 3.5 mmHg (1 week), 16.7 ± 3.5 mmHg (1 month), 16.5 ± 2.6 mmHg (2 months), and 16.1 ± 2.3 mmHg (3 months) in the supine position, and 21.6 ± 3.7 mmHg (1 week), 20.1 ± 4.4 mmHg (1 month), 19.8 ± 2.9 mmHg (2 months), and 19.2 ± 2.5 mmHg (3 months) in DLDP. Compared to the baseline values, the IOP in each position was significantly decreased at every time point of the follow-up period (all *P* < 0.001). The IOP in the supine position and DLDP was significantly higher than that in the sitting position at every time point of the follow-up period (all *P* < 0.001). The IOP in DLDP was significantly higher than that in the supine position at every time point of the follow-up period (all *P* < 0.001, Fig 1).

Table 2 summarizes the mean percentage of IOP reduction in each position during the follow-up period. There was no significant difference between the sitting and supine positions. However, the mean percentage of IOP reduction in DLDP was significantly lower than that in the sitting position at every time point of the follow-up period (1 week, *P* = 0.001; 1 month, *P* = 0.015; 2 months, *P* < 0.001; 3 months, *P* = 0.003) and that in the supine position (1 week, *P* = 0.015; 1 month, *P* = 0.009; 2 months, *P* = 0.005; 3 months, *P* = 0.006).

**Table 2:** Mean Percentage of Intraocular Pressure Reduction in the Different Body Position.

Time point	IOP reduction, %			*P* value*		
	Sitting	Supine	DLDP	*P1*	*P2*	*P3*
1 week	17.7 ± 13.4 (-16.8–39.7)	15.4 ± 14.8 (-14.4–44.4)	8.4 ± 8.5 (-12.0–22.1)	0.115	0.001	0.015
1 month	22.3 ± 16.1 (-8.6–52.9)	21.0 ± 14.5 (-5.2–53.7)	14.4 ± 17.1 (-20.6–43.2)	0.380	0.015	0.009
2 month	24.2 ± 14.2 (-8.9–52.7)	21.1 ± 12.9 (-4.7–49.6)	15.0 ± 14.4 (-20.9–39.1)	0.068	<0.001	0.005
3 month	25.1 ± 14.4 (-2.3–52.7)	22.8 ± 12.4 (-4.7–51.6)	17.0 ± 15.4 (-20.9–45.2)	0.195	0.003	0.006

DLDP, dependent lateral decubitus position; IOP, intraocular pressure; *P1*, sitting vs supine; *P2*, sitting vs DLDP; *P3*, supine vs DLDP. Data were expressed as mean ± standard deviation (range).

* Paired *t*-test with adjustment for multiple comparisons by Bonferroni correction.

During the follow-up period, the extent of IOP differences between the sitting position and supine position, sitting position and DLDP, and supine position and DLDP did not change significantly (Table 3).

**Table 3:** The Intraocular Pressure Differences Between Body Positions.

N = 30	Sitting vs supine, mmHg	P*	Sitting vs DLDP, mmHg	P*	Supine vs DLDP, mmHg	P*
Baseline	2.1 ± 1.5 (0.1–5.1)		4.8 ± 3.0 (0.6–12.2)		2.6 ± 2.7 (-2.4–7.1)	
1 week	2.2 ± 1.4 (0.4–5.6)	0.778	6.0 ± 2.4 (0.5–9.5)	0.055	3.8 ± 2.7 (-2.4–7.6)	0.051
1 month	1.9 ± 1.7 (-0.4–6.0)	0.469	5.4 ± 3.0 (-0.6–13.7)	0.460	3.5 ± 2.1 (-0.2–9.1)	0.226
2 month	2.2 ± 1.4 (-0.2–4.4)	0.865	5.5 ± 1.4 (2.9–7.9)	0.237	3.3 ± 1.8 (-0.9–6.2)	0.227
3 month	2.0 ± 1.7 (-0.3–5.0)	0.758	5.1 ± 1.6 (2.7–8.2)	0.591	3.1 ± 1.8 (-0.1–7.5)	0.385

DLDP, dependent lateral decubitus position; IOP, intraocular pressure. Data were expressed as mean ± standard deviation (range).

* Compare to baseline value. Repeated measures analysis of variance followed by Bonferroni post-hoc test.

## Discussion

IOP is dynamic and can change continuously in different situations. Prior studies have noted the importance of IOP fluctuations in the development and progression of glaucoma [5,7]. In addition, although some controversies exists, the extent of the posture-induced IOP changes is greater in glaucoma patients than in normal subjects [34–38]. Therefore, measuring IOP in a sitting position might be misleading for judging the therapeutic effectiveness, whereas measuring IOP in various positions may play a considerable role in management of glaucoma.

Prior to ALT, an apparent increase in IOP was noted in our study according to postural changes from the sitting position to supine position and DLDP. These results agree with the findings of recent studies, which found that the IOP was significantly increased when the patients changed their position from sitting to supine or DLDP [9,34,39]. The exact mechanism of posture-induced IOP change has not been determined yet. With regard to the supine position-induced IOP elevation, several assumptions have been made, including an increase in episcleral venous pressure and ophthalmic artery pressure, and alteration in the rate of uveoscleral outflow due to increased choroidal blood volume [40–42]. Likewise, an increase in episcleral venous pressure and engorgement of the choroidal vascular bed caused by redistribution of body fluids in the recumbent position was suggested as a mechanism of DLDP-induced IOP elevation [11,43].

Our results show that ALT has a limited effect on posture-induced IOP changes. This finding is consistent with previous research, which showed that ALT had little effect on postural behavior of IOP [26]. In contrast, according to a study by Sawada et al.[21], trabeculectomy significantly reduces the extent of posture-induced IOP changes by yielding an alternative aqueous pathway through the filtering bleb independent of the conventional trabecular meshwork outflow pathway. Two main hypotheses aim to explain the mechanism of facilitation of the aqueous outflow by ALT: one hypothesis postulates that mechanical contraction of the trabecular meshwork by thermal energy opens the intratrabecular space, whereas the other one assumes that cellular activation of the trabecular meshwork causes remodeling of the extracellular matrix [44–46]. Therefore, promoting aqueous outflow in compromised trabecular meshwork by these mechanisms might explain why ALT (in contrast to trabeculectomy) has little effect on posture-induced IOP changes.

In the present study, ALT has a favorable IOP-lowering effect in the sitting position, supine position, and DLDP. However, the mean percentage of IOP reduction in DLDP was significantly lower than those in the sitting or supine position during the follow-up period. The mean percentage of IOP reduction in the sitting position was similar to that observed in a previous study conducted on Korean OAG patients, which found a 26.3% IOP reduction at 3 months after ALT [47]. However, it is also important to note that the mean percentage of IOP reduction in DLDP in our study was not sufficient for successful ALT, which usually indicates an IOP reduction of ≥ 20% of the pretreatment value [48]. The exact mechanism regarding the higher IOP in DLDP than in the supine position is little known. Previous studies have suggested that IOP elevation in DLDP, compared to supine position, might have been attributed by the greater increase of episcleral venous pressure due to the greater compression of the neck vessels by neck flexion or compression by pillow [11,30,34,39]. In addition, as the level of eyeball in DLDP is lower than in the supine position concerning the heart level, the increase in mean ophthalmic arterial pressure might have also attributed to the elevation of IOP [15,49]. Hence, we speculate that the effect of episcleral venous pressure or choroidal vascular volume on IOP might be greater in the DLDP than in the supine position. Because the mechanism of ALT does not involve bypassing the compromised trabecular meshwork, still dependent on the conventional outflow pathway, it might have a limited effect reducing pressure that could compensate greater IOP elevation in DLDP compared with supine position [21]. Considering aforementioned finding, the eyes in DLDP would be more subjective to the rise of IOP that might counteract the IOP lowering effect of ALT. However, further studies regarding the measurements of episcleral venous pressure or ophthalmic arterial pressure are essential to clarify this issue. In a subgroup of patients who manifest significant IOP elevation in DLDP which might affect patient’s disease progression, other treatment modality yielding alternate aqueous pathway such as trabeculectomy could be more useful.

Based on the previous study, one might question whether the change in original IOP level at the sitting position would affect the degree of posture-induced IOP fluctuation [50]. However, other studies, such as the study of Armaly et al., [51] have shown no significant differences in posture-induced IOP elevation regarding the original IOP level. In addition, it has been reported that there was no significant relationship between the low original IOP and the posture-induced IOP change, including the study of ocular hypotensive medical treatment in NTG patients, and surgical treatment using trabeculectomy in OAG patients [21,38,52]. Similarly, despite the decrease of IOP in the sitting position after treatment, the magnitude of posture-induced IOP changes (sitting position vs. supine, 1.9–2.2 mmHg; sitting position vs. DLDP, 5.1–6.0 mmHg) were not significantly correlated with the level of IOPs in sitting position in each follow up periods (data not shown). Additionally, the magnitude of posture-induced IOP changes were comparable to the previous reports, which have demonstrated 1.2–3.3 mmHg increase in supine position, and 4.5–5.4 mmHg increase in DLDP compared with sitting position measured by rebound tonometer in OAG patients [10,38]. Therefore, it is less likely that original IOP in the sitting position had significantly affected the degree of posture-induced IOP fluctuation.

To our knowledge, this study is the first to assess the effect of the ALT in DLDP. However, this study has several limitations that need to be acknowledged. First, the sample size was small and study subjects used ocular hypotensive medications during the study. Moreover, we did not assess the effect of adherence of hypotensive medication which could have influenced the variation of IOP. However, in the previous studies, the use of hypotensive ophthalmic solution had no significant effect on posture-induced IOP changes in glaucoma patients [21,52]. Second, the short duration of each position (10 min) may be another limitation. As indicated by Lee et al [39]., maintaining each position for a longer time may produce different results. Third, as the study was conducted in a day time, the current findings may not reflect accurately the real physiological and environmental sleeping status. Fourth, we did not assess the posture-induced changes in ocular perfusion and cerebrosopinal fluid pressure which might have a compensatory role in IOP variation. Another question is the accuracy of the IOP measurements by rebound tonometry in different positions. However, it has been demonstrated that the accuracy of rebound tonometry is comparable with that of tonopen and GAT, and also our results have shown strong correlation between two measurements [53–55]. Lastly, the IOP measurements could have been subject to regression to the mean. However, the effect of regression to the mean is speculated to be small since we measured IOP multiple times (3 consecutive sets; 6 measurements for each set) and averaged [56].

In conclusion, our study shows that ALT is effective in lowering the IOP in various body positions. However, the degree of the IOP-lowering effect differs depending on the position and is significantly lower in DLDP than in the sitting and supine positions. In addition, ALT had a limited effect on the extent of posture-induced IOP changes in OAG. Our findings should be considered when treating OAG with ALT.
